# Comprehensive Comparison of MOCVD- and LPCVD-SiN_x_ Surface Passivation for AlGaN/GaN HEMTs for 5G RF Applications

**DOI:** 10.3390/mi14112104

**Published:** 2023-11-16

**Authors:** Longge Deng, Likun Zhou, Hao Lu, Ling Yang, Qian Yu, Meng Zhang, Mei Wu, Bin Hou, Xiaohua Ma, Yue Hao

**Affiliations:** 1 State Key Discipline Laboratory of Wide Bandgap Semiconductor Technology, School of Microelectronics, Xidian University, Xi’an 710071, China; 2 Advanced Materials and Nanotechnology, Xidian University, Xi’an 710071, China

**Keywords:** AlGaN/GaN, high electron mobility transistors (HEMTs), SiN_x_ passivation, low-pressure chemical vapor deposition (LPCVD), ohmic contact, SiN_x_/GaN interface

## Abstract

Passivation is commonly used to suppress current collapse in AlGaN/GaN HEMTs. However, the conventional PECV-fabricated SiN_x_ passivation layer is incompatible with the latest process, like the “passivation-prior-to-ohmic” method. Research attention has therefore turned to high-temperature passivation schemes. In this paper, we systematically investigated the differences between the SiN_x_/GaN interface of two high-temperature passivation schemes, MOCVD-SiN_x_ and LPCVD-SiN_x_, and investigated their effects on the ohmic contact mechanism. By characterizing the device interface using TEM, we reveal that during the process of MOCVD-SiN_x_, etching damage and Si diffuses into the semiconductor to form a leakage path and reduce the breakdown voltage of the AlGaN/GaN HEMTs. Moreover, N enrichment at the edge of the ohmic region of the LPCVD-SiN_x_ device indicates that the device is more favorable for TiN formation, thus reducing the ohmic contact resistance, which is beneficial to improving the PAE of the device. Through the CW load-pull test with drain voltage *V*_DS_ = 20V, LPCVD-SiN_x_ devices obtain a high PAE of 66.35%, which is about 6% higher than MOCVD-SiN_x_ devices. This excellent result indicates that the prospect of LPCVD-SiN_x_ passivation devices used in 5G small terminals will be attractive.

## 1. Introduction

With the rapid growth of mobile data volume, traditional 4G networks are no longer able to meet the demands for higher speeds, lower latency, and more reliable connections. As a result, the development of 5G as the next generation of mobile communication technology aims to provide more efficient, flexible, and innovative communication solutions to support various novel applications and the expanding needs of the digital society. In 5G applications, GaN semiconductors offer unique advantages including wide bandgap and high operation frequency compared to Si and group III-V material [1]. Devices based on GaN materials exhibit higher output power density and energy conversion efficiency, enabling system miniaturization and lightweight design. Today, GaN devices have been widely used in radio frequency (RF) applications [2,3,4] and power electronics [5,6,7]. However, during the process of GaN HEMT epitaxial growth, surface states like dangling bonds or defects will exist on the interrupted surfaces [8,9]. These surface states will lead to some adverse phenomena such as current collapse, thereby significantly impacting the RF output performance of GaN HEMT devices [10,11]. There are currently various methods available for suppressing surface states, such as growth-controlling conditions [12,13] or surface passivation [14]. Surface passivation has become an effective and common method due to its simplicity. It has a relatively simple process and can reduce the possibility of chemical contamination or mechanical damage to the surface during subsequent packaging processes. The passivation material mostly used on GaN HEMTs is SiN_x_ [15] and the most commonly used deposition method for it is plasma-enhanced chemical vapor deposition (PECVD) with a growth temperature below 350 °C [16]. However, due to its tendency to crack under high-temperature conditions [17], it is not possible to carry out the “passivation-prior-to-ohmic” process [18]. In addition, SiN_x_ growth using PECVD has lower film compactness compared to the SiN_x_ deposited by metal organic chemical vapor deposition (MOCVD) and low-pressure chemical vapor deposition (LPCVD) [18,19]. The active plasma sources in PECVD can also potentially cause damage to the surface of AlGaN or GaN and further degrade the quality of the deposited SiN_x_ itself [20]. Therefore, improved high-temperature solutions, including MOCVD (over 900 °C) and LPCVD (over 750 °C), are used for depositing the passivation layer [21,22,23]. MOCVD, an in-situ SiNx passivation technique, is employed in which an epitaxial layer is deposited using MOCVD, followed by the deposition of an additional SiNx passivation layer within the same chamber. This in-situ growth approach helps to prevent some negative impacts during the process chamber transfer, such as oxidation reactions. During the LPCVD process for SiNx deposition, the pressure is typically maintained at around 200 mTorr. At a specific temperature, the lower operating pressure results in an increased mean free path of gas molecules within the chamber, leading to a significant reduction in diffusion rate. Consequently, the reaction time is prolonged, thereby achieving the objective of enhancing thin film quality and density. The research above has revealed that SiN_x_ passivation layers deposited at high temperatures exhibit higher thermal stability and better growth quality, which can significantly suppress surface state density and enhance the output characteristics of devices.

However, it is noted that a high-temperature deposited SiN_x_ layer is often used in power electronic devices to form MIS gate structures [23,24], while there are limited evaluations in the field of RF applications [25] and there has been little research on the interface under SiN_x_ layers and the mechanism of ohmic contact, which are important concerns for enhancing the RF performance of AlGaN/GaN HEMTs. The impact of the passivation layer on ohmic alloying is not yet clear, and there is no relevant research on the interface quality of the passivation layer and its interdiffusion with the barrier layer. Therefore, this work systematically compares MOCVD-SiN_x_ and LPCVD-SiN_x_, which are known for their high-temperature processes to evaluate interface quality and the impact on ohmic contact. Finally, this study will assess the performance between the two passivation layer approaches in RF applications.

## 2. Device Structure and Fabrication

**Figure 1:**
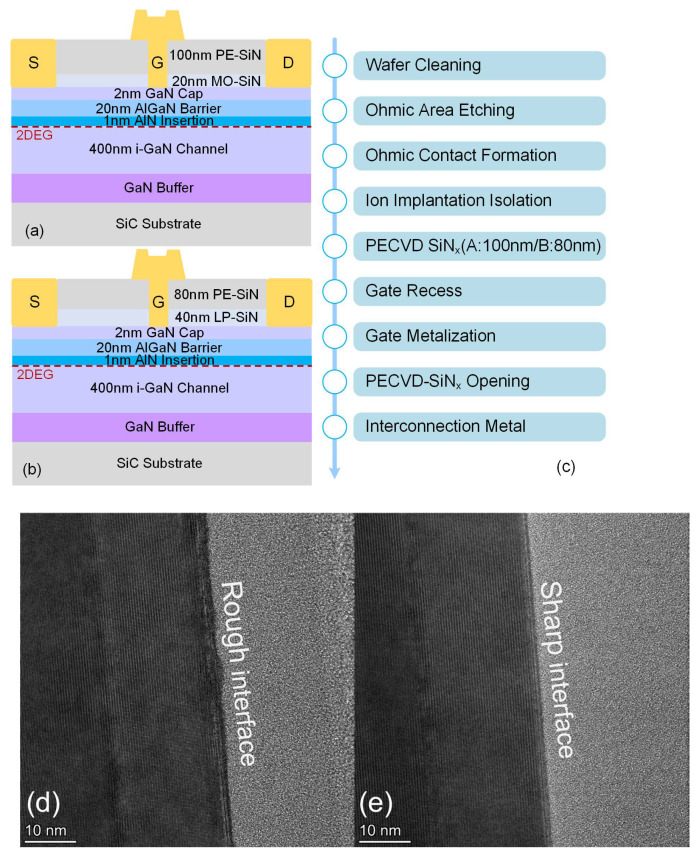
The device structure diagram of (**a**) sample A and (**b**) sample B. (**c**) The fabrication process flow of the samples. The TEM image of the interface quality of SiN_x_/GaN in (**d**) sample A and (**e**) sample B.

The structures of sample A and sample B used in this work are shown in Figure 1a,b. The AlGaN/GaN heterostructure of both samples was grown on a three-inch SiC substrate using MOCVD. The epilayer, from the bottom to the top, included a GaN buffer (a 400 nm unintentionally doped GaN channel layer). On top of the GaN channel layer, there was a 1 nm AlN insertion layer, facilitating the growth of a 20 nm AlGaN layer with 25% Al composition. A 2 nm GaN cap layer was deposited on the barrier layer. Considering the growth quality of the passivation layer and the stress management, the difference between sample A and sample B is that there was a 20 nm in situ SiN_x_ passivation layer utilized using MOCVD and a 40 nm ex situ SiN_x_ passivation layer utilized by LPCVD. The device fabrication process followed the “passivation-prior-to-ohmic” strategy. The fabrication process flow is shown in Figure 1c. The SiN_x_ passivation layer above the ohmic region was first etched away by F-based etching. The F-based etching gas mixture consists of CF4/O2 = 25/5 sccm, and the etching pressure is set to 5 mTorr. Then, another ohmic photolithography process was performed followed by ohmic contact formation. The ohmic contact metal stack was Ti/Al/Ni/Au = 20/160/45/55 nm, with annealing performed at 860 °C/60 s under ambient N_2_ conditions. The electrical isolation of the devices was achieved using nitrogen ion implantation. To form the T-gate structure, on sample A, the 20 nm SiN_x_ passivation layer was supplemented with 100 nm SiN_x_ grown by PECVD, and the 40 nm SiN_x_ layer was supplemented with an additional 80 nm SiN_x_ grown by PECVD on sample B. The T-gate electrode was formed by Ni/Au = 45/400 nm stacks with a 0.5 μm foot length and a 1 μm cap length, while the gate width was 100 μm. Finally, interconnection metal fabrication was completed by evaporating Ti/Au = 20/400 nm.

## 3. Results and Discussion

After the fabrication, the samples were tested using a Keysight B1500A semiconductor parameter analyzer to obtain the result of the transmission line model (TLM) and the DC characteristics. The pulse I-V characteristics were tested using a Keithley 4200A-SCS parameter analyzer.

A. The Epitaxial Growth of Device

Figure 1d,e compare the epitaxial growth quality of sample A and sample B after ohmic annealing. It can be observed from the transmission electron microscopy (TEM) that Si was significantly diffused at the interface of sample A, resulting in a rough SiN_x_/GaN boundary. In contrast, in sample B, there was a clear boundary between the LPCVD-SiN_x_ and the semiconductor interface, indicating the good suppression of Si diffusion. This is because, during the process of depositing the SiN_x_ film using MOCVD, SiH_4_ had an etching effect on the semiconductor layer, allowing Si to diffuse into the semiconductor as an impurity. According to the results, we suppose that LPCVD can effectively prevent the diffusion of Si. Zhu et al. have demonstrated that the surface diffusion of Si is insufficient to compensate for the electron concentration and enhance performance [26]. On the contrary, etching damage accompanied by the diffusion of Si into the semiconductor will generate a leakage current path through the gate, affecting the reverse characteristics of the device [27].

B. Formation Mechanism of Ohmic Contact

**Figure 2:**
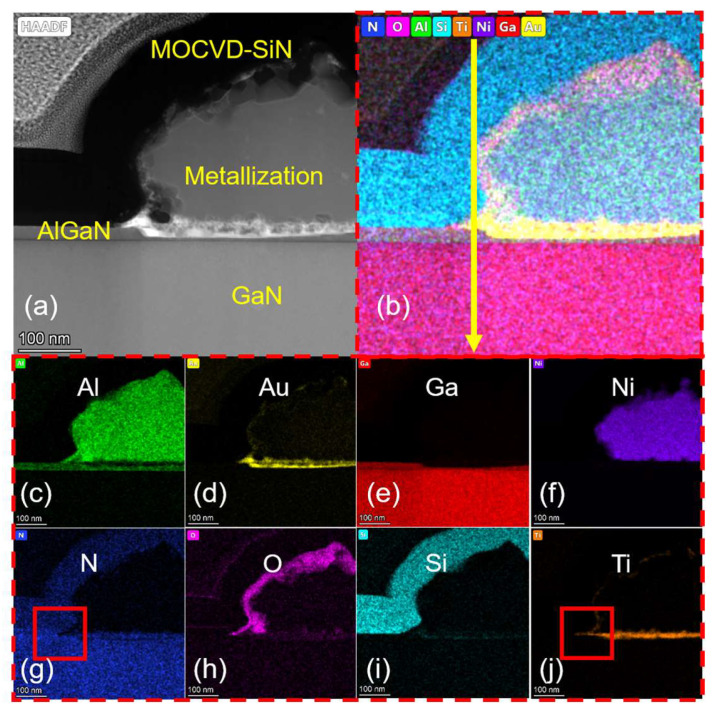

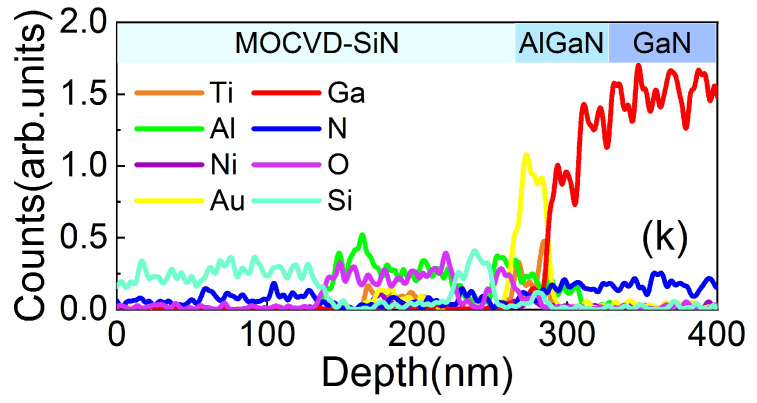
(**a**) The HAADF-STEM micrograph of the sidewall ohmic region for sample A. (**b**) EDX mapping of all elements. (**c**–**j**) EDX mapping of Al, Au, Ga, Ni, N, O, Si, and Ti. (**k**) EDS line scan profile of yellow arrow presented in (**b**).

**Figure 3:**
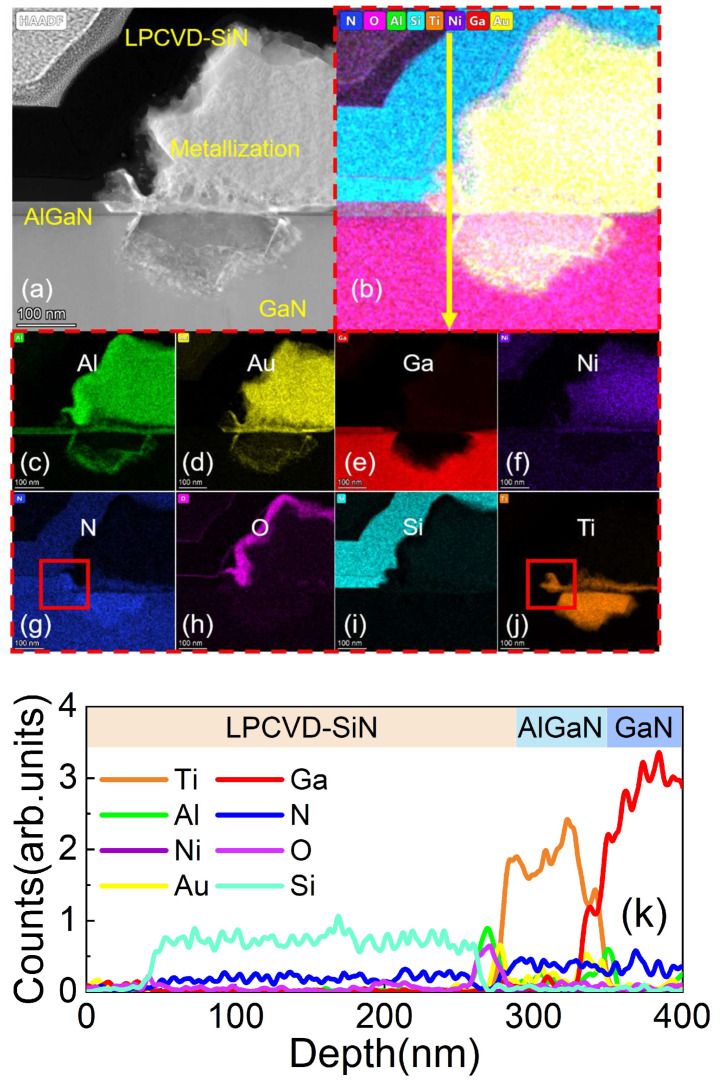
(**a**) The HAADF-STEM micrograph of the sidewall ohmic region for sample B. (**b**) EDX mapping of all elements. (**c**–**j**) EDX mapping of Al, Au, Ga, Ni, N, O, Si, and Ti. (**k**) EDS line scan profile of yellow arrow presented in (**b**).

Figure 2 and Figure 3 compare the morphology of ohmic regions in samples A and B. The formation of TiN in high-temperature annealing can promote ohmic metal formation by ensuring direct contact with 2DEG or by forming N vacancies reported by many reports [28,29,30,31]. There was no apparent low-work function TiN alloy incorporation in the edge of the ohmic region of sample A, while there was large TiN alloy incorporation in sample B. Since there was sidewall contact between the ohmic metal stack and the passivation layer in the “passivation-prior-to-ohmic” scheme, some different alloy reactions occurred under the same annealing conditions. Based on an analysis of the distribution of Ti and N elements at the edge of the ohmic region for sample A (shown in the red box in Figure 2g,j) and sample B (shown in the red box in Figure 3g,j), we infer that this is because the deposition temperature of MOCVD-SiN_x_ was above 900 °C while the annealing temperature of sample A was lower than 900 °C, resulting in N being difficult to participate in the alloy reaction in order to form TiN from the side walls during the annealing alloying process. In contrast, the deposition temperature of LPCVD-SiN_x_ was 780 °C, which is less than the annealing temperature of 860 °C. This may make it easier for N to combine with Ti to form TiN in sample B. This enrichment of TiN at the edge of the ohmic region, which is also compared in Figure 2k and Figure 3k, effectively reduced the ohmic contact resistance.

C. Ohmic Contact Resistance and DC I-V Characteristics

**Figure 4:**
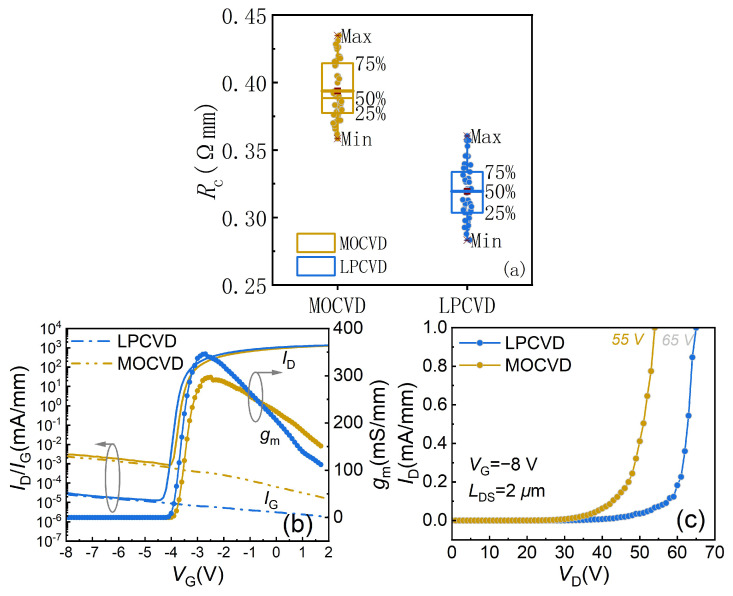
(**a**) TLM result of the MOCVD-SiN_x_ and LPCVD-SiN_x_ device. (**b**) Transfer I–V characteristics in semi-log scale of the MOCVD-SiN_x_ and LPCVD-SiN_x_ device. (**c**) Breakdown characteristics of the MOCVD-SiN_x_ and LPCVD-SiN_x_ devices.

The ohmic contact results obtained by the transmission line model (TLM) test are shown in Figure 4a. The ohmic contact resistance (*R*_c_) for sample B with apparent side wall effect was 0.32 ± 0.05 Ω·mm and the sheet resistance (*R*_sh_) was 291 Ω/sq, whereas the *R*_c_ for sample A was 0.39 ± 0.05 Ω·mm, with an *R*_sh_ of 302 Ω/sq. Figure 4b displays the DC characteristic curves of samples A and B. From the transfer I–V curves, it can be observed that the threshold voltage for sample A was −3.7 V with a maximum transconductance (*g*_m,max_) of 294 mS/mm, while sample B had a threshold voltage of −3.8 V and *g*_m,max_ of 346 mS/mm, which was 50 mS/mm higher than sample A.

Under the conditions of gate voltage (*V*_gs_) = 2 V and drain voltage (*V*_ds_) = 10 V, the maximum output currents were 1.28 A/mm and 1.34 A/mm for samples A and B, respectively. To minimize the influence of thermal resistance on on-resistance (*R*_on_), the pulse test at a quiescent voltage of (0 V, 0 V) was performed with a 2 μm drain source spacing (*L*_DS_) device. The measured *R*_on_ value for sample A was 1.75 Ω·mm and that for sample B was 1.54 Ω·mm. It can be seen that sample B had a lower *R*_on_, which is consistent with the superior TLM results on sample B. Moreover, sample B, which lacks the diffusion of Si and etching damage, did not form an additional leakage path in passivation layers, resulting in relatively lower gate leakage current by 1–2 orders of magnitude below 10^−5^ mA/mm compared to sample A.

Figure 4c depicts the breakdown characteristics for the samples. At *V*_gs_ = −8 V, the breakdown voltage of sample A was measured to be 55 V for a 2 μm *L*_DS_ device, while the breakdown voltage of sample B was 65 V. This indicates that the excellent growth interface of LPCVD-SiN_x_ can generate a smoother electric field, thereby enhancing the breakdown characteristics of the device.

D. Pulsed I-V Characteristics

**Figure 5:**
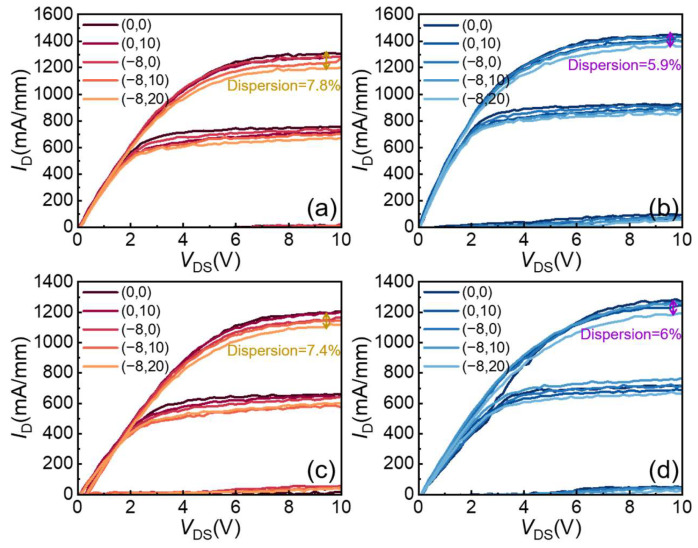
Pulsed output characteristic of (**a**) sample A and (**b**) sample B at 25 °C. Pulsed output characteristics of (**c**) sample A and (**d**) sample B at 85 °C.

In RF applications, especially for an RF power output, the temperature of the working environment usually increases due to device heat dissipation. Therefore, to characterize the stability of the passivation layer, pulse I-V measurements were performed at room temperature of 25 °C and high temperature of 85 °C with a pulse width and a duty cycle of 500 ns and 0.05%, respectively. The voltage range of gate voltage (*V*_GS_) was −4 to 2 V with a step of 3 V. The gate and drain quiescent bias (*V*_GQ_ and *V*_DQ_) used for the pulse test is shown in Figure 5, ranging from (*V*_GQ_, *V*_DQ_) = (0 V, 0 V)~(−8 V, 20 V). Under room temperature conditions, the current collapse shown in Figure 5a of sample A was 7.8% for the high reverse gate voltage and high drain voltage condition of (−8 V, 20 V), while that of sample B shown in Figure 5b was 5.9%. At 85 °C, the current collapse of sample A was 7.4% (Figure 5c), while that of sample B was 6% (Figure 5d). Comparing the passivation effects between high and low temperatures, the variations observed were relatively insignificant and we thought that the discrepancy was a consequence of different surface trapping states at the interface to the SiN layer. The different growth methods and recipes for the passivation layer had a significant impact on the introduction of traps [32,33], and, in the future, we will focus on more accurate trap testing, like deep-level transient spectroscopy (DLTS), to demonstrate the differences in traps related to the two passivation layers. Nevertheless, both samples show excellent thermal stability and also show that sample B has a better passivation effect at either room temperature or at high temperatures. These results prove that the high-temperature process of SiN_x_ deposition is beneficial to improving the thermal stability of the passivation.

E. Small- and Large Signal Power Characteristic

**Figure 6:**
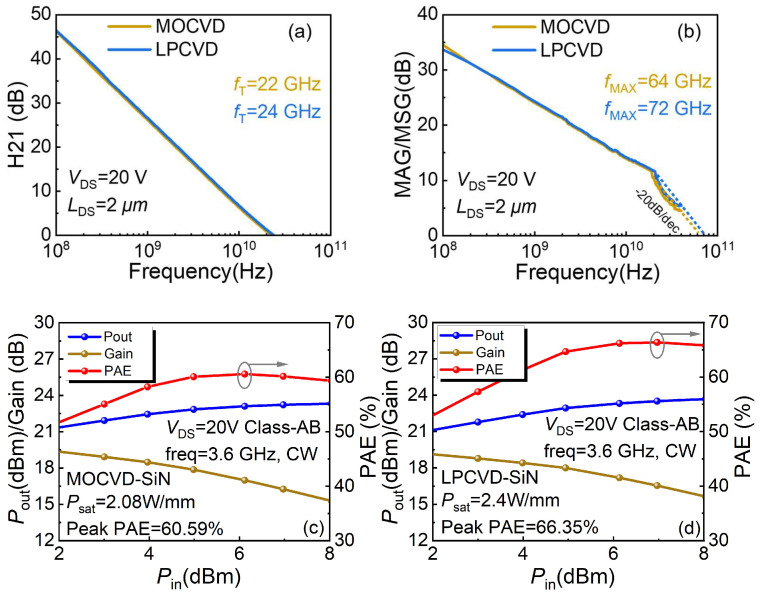
Small signal comparison between MOCVD- and LPCVD-SiN_x_ of (**a**) *f*_T_ and (**b**) *f*_max_. Large-signal results of (**c**) the MOCVD-SiN_x_ device and (**d**) the LPCVD-SiN_x_ device.

Small-signal measurements of the two samples are shown in Figure 6a,b. S-parameters were measured from 0.1 GHz to 40 GHz using a Keysight network analyzer. The current gain cutoff frequency *f*_T_ for sample A at *V*_ds_ = 20 V was 22 GHz, while that of sample B was 24 GHz. The maximum oscillation frequency *f*_max_ was 64 GHz in sample A and 72 GHz in sample B. The improvement of *f*_max_ may be ascribed to the better transconductance and reduced parasitic resistance deriving from low *R*_c_ in sample B [34].

**Figure 7:**
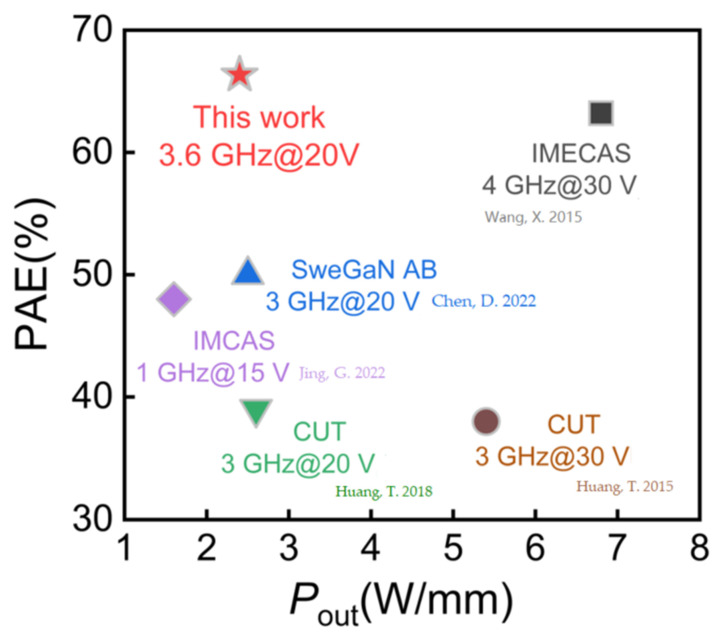
Benchmark of sub-6G high-signal power characteristics for state-of-the-art AlGaN/GaN HEMTs [18,25,36,37,38] .

CW load–pull measurements were measured at 3.6 GHz using a Focus load–pull system with class-AB bias. The impedance matching in the load-pull test is PAE optimum tuning. These results are shown in Figure 6c,d. It can be seen that sample B has better output power density and power-added efficiency (PAE). This is attributed to lower leakage current [35] and lower parasitic resistance [34] in sample B. According to the large-signal test, sample B exhibited a PAE of 66.35% under a drain voltage bias of 20 V, which is 6% higher than 60.59% of sample A. The sample B fabricated in this study exhibited significant efficiency advantages in low-voltage power output compared to other state-of-the-art LPCVD-SiN_x_ AlGaN/GaN HEMTs measured at a sub-6G condition, as shown in Figure 7 [18,25,36,37,38].

## 4. Conclusions

Due to the low-temperature characteristics of PECVD-SiNx, it cannot meet the requirements of an advanced “passivation-prior-to-ohmic” process. This study investigates SiNx passivation fabricated using two high-temperature deposition methods. In this paper, we compared the differences between MOCVD-SiN_x_ and LPCVD-SiN_x_ in terms of ohmic contact and related interface. We discovered that the growth interface of LPCVD-SiN_x_ was smoother than that of MOCVD-SiN_x_, resulting in better leakage suppression. Additionally, LPCVD-SiN_x_ devices achieved improved RF output performance by forming lower Ohmic contact resistance. The maximum current of the LPCVD-SiN_x_ device exceeded 1.3 A/mm at *V*_gs_ = 2 V, with gate leakage below 10^−5^ mA/mm. The *f*_max_ of the 2 μm *L*_DS_ device reached 72 GHz. Under a drain voltage of 20 V, the output power exceeded 2.4 W/mm with PAE greater than 66.35%. The results presented in this study demonstrates significant efficiency advantages in low-voltage power output compared to other state-of-the-art LPCVD-SiNx AlGaN/GaN HEMT operated at 5G frequency spectrum. These results demonstrate the excellent performance of LPCVD-SiN_x_ devices in small-sized modules working in low-voltage applications and highlight its prospects in 5G small terminals.

## References

[1] Hao Y., Ma X., Mi M., Yang L. (2021). Research on GaN-Based RF Devices: High-Frequency Gate Structure Design, Submicrometer-Length Gate Fabrication, Suppressed SCE, Low Parasitic Resistance, Minimized Current Collapse, and Lower Gate Leakage. IEEE Microw. Mag..

[2] Mishra U.K., Parikh P., Wu Y.F. (2002). AlGaN/GaN HEMTs—An Overview of Device Operation and Applications. Proc. IEEE.

[3] Lu H., Hou B., Yang L., Zhang M., Deng L., Wu M., Si Z., Hunag S., Ma X., Hao Y. (2022). High RF performance GaN-on-Si HEMTs with passivation implanted termination. IEEE Electron Device Lett..

[4] Wu M., Zhang M., Yang L., Hou B., Yu Q., Li S., Shi C., Zhao W., Lu H., Chen W. First Demonstration of State-of-the-art GaN HEMTs for Power and RF Applications on A Unified Platform with Free-standing GaN Substrate and Fe/C Co-doped Buffer. Proceedings of the 2022 International Electron Devices Meeting (IEDM).

[5] Wang Z., Zhang Z., Wang S., Chen C., Wang Z., Yao Y. (2019). Design and optimization on a novel high-performance ultra-thin barrier AlGaN/GaN power HEMT with local charge compensation trench. Appl. Sci..

[6] Wang Z., Yang D., Cao J., Wang F., Yao Y. (2019). A novel technology for turn-on voltage reduction of high-performance lateral heterojunction diode with source-gate shorted anode. Superlattices Microstruct..

[7] Chen K.J., Häberlen O., Lidow A., lin Tsai C., Ueda T., Uemoto Y., Wu Y. (2017). GaN-on-Si power technology: Devices and applications. IEEE Trans. Electron Devices.

[8] Mitrofanov O., Manfra M. (2003). Mechanisms of gate lag in GaN/AlGaN/GaN high electron mobility transistors. Superlattices Microstruct..

[9] Makiyama K., Ohki T., Okamoto N., Kanamura M., Masuda S., Nakasha Y., Joshin K., Imanishi K., Hara N., Ozaki S. (2011). High-power GaN-HEMT with low current collapse for millimeter-wave amplifier. Phys. Status Solidi C.

[10] Huang S., Jiang Q., Yang S., Zhou C., Chen K.J. (2012). Effective passivation of AlGaN/GaN HEMTs by ALD-grown AlN thin film. IEEE Electron Device Lett..

[11] Hashizume T., Hasegawa H. (2004). Effects of nitrogen deficiency on electronic properties of AlGaN surfaces subjected to thermal and plasma processes. Appl. Surf. Sci..

[12] Chen J., Qin J., Xiao W., Wang H. (2022). Effective Suppression of Current Collapse in AlGaN/GaN HEMT With N_2_O Plasma Treatment Followed by High Temperature Annealing in N 2 Ambience. IEEE J. Electron Devices Soc..

[13] Weimann N.G., Manfra M.J., Wachtler T. (2003). Unpassivated AlGaN-GaN HEMTs with minimal RF dispersion grown by plasma-assisted MBE on semi-insulating 6H-SiC substrates. IEEE Electron Device Lett..

[14] Ohno Y., Nakao T., Kishimoto S., Maezawa K., Mizutani T. (2004). Effects of surface passivation on breakdown of AlGaN/GaN high-electron-mobility transistors. Appl. Phys. Lett..

[15] Zhang Y., Huang S., Wei K., Zhang S., Wang X., Zheng Y., Liu G., Chen X., Li Y., Liu X. (2020). Millimeter-wave AlGaN/GaN HEMTs with 43.6% power-added-efficiency at 40 GHz fabricated by atomic layer etching gate recess. IEEE Electron Device Lett..

[16] Pei Y., Rajan S., Higashiwaki M., Chen Z., DenBaars S.P., Mishra U.K. (2009). Effect of dielectric thickness on power performance of AlGaN/GaN HEMTs. IEEE Electron Device Lett..

[17] Moon S.W., Lee J., Seo D., Jung S., Choi H.G., Shim H., Yim J.S., Twynam J., Roh S.D. (2014). High-voltage GaN-on-Si hetero-junction FETs with reduced leakage and current collapse effects using SiNx surface passivation layer deposited by low pressure CVD. Jpn. J. Appl. Phys..

[18] Wang X., Huang S., Zheng Y., Wei K., Chen X., Liu G., Yuan T., Luo W., Pang L., Jiang H. (2015). Robust SiN x/AlGaN interface in GaN HEMTs passivated by thick LPCVD-grown SiN x layer. IEEE Electron Device Lett..

[19] Siddique A., Ahmed R., Anderson J., Nazari M., Yates L., Graham S., Holtz M., Piner E.L. (2019). Structure and interface analysis of diamond on an AlGaN/GaN HEMT utilizing an in situ SiN x interlayer grown by MOCVD. ACS Appl. Electron. Mater..

[20] Zhang S., Wei K., Ma X.H., Hou B., Liu G.G., Zhang Y.C., Wang X.-H., Zheng Y.-K., Huang S., Li Y.-K. (2019). Reduced reverse gate leakage current for GaN HEMTs with 3 nm Al/40 nm SiN passivation layer. Appl. Phys. Lett..

[21] Lu X., Ma J., Jiang H., Liu C., Lau K.M. (2014). Low trap states in in situ SiNx/AlN/GaN metal-insulator-semiconductor structures grown by metal-organic chemical vapor deposition. Appl. Phys. Lett..

[22] Lu H., Hou B., Yang L., Niu X., Si Z., Zhang M., Wu M., Mi M., Zhu Q., Cheng K. (2021). Aln/GaN/InGaN coupling-channel HEMTs for improved g m and gain linearity. IEEE Trans. Electron Devices.

[23] Hua M., Liu C., Yang S., Liu S., Fu K., Dong Z., Cai Y., Zhang B., Chen K.J. (2015). GaN-based metal-insulator-semiconductor high-electron-mobility transistors using low-pressure chemical vapor deposition SiN x as gate dielectric. IEEE Electron Device Lett..

[24] Jiang H., Liu C., Chen Y., Lu X., Tang C.W., Lau K.M. (2017). Investigation of in situ SiN as gate dielectric and surface passivation for GaN MISHEMTs. IEEE Trans. Electron Devices.

[25] Huang T., Malmros A., Bergsten J., Gustafsson S., Axelsson O., Thorsell M., Rorsman N. (2015). Suppression of dispersive effects in AlGaN/GaN high-electron-mobility transistors using bilayer SiN x grown by low pressure chemical vapor deposition. IEEE Electron Device Lett..

[26] Zhu L., Zhou Q., Chen K., Gao W., Cai Y., Cheng K., Li Z., Zhang B. (2022). The Modulation Effect of LPCVD-Si x N y Stoichiometry on 2-DEG Characteristic of UTB AlGaN/GaN Heterostructure. IEEE Trans. Electron Devices.

[27] Song L., Fu K., Zhang Z., Sun S., Li W., Yu G., Hao R., Fan Y., Shi W., Cai Y. (2017). Interface Si donor control to improve dynamic performance of AlGaN/GaN MIS-HEMTs. AIP Adv..

[28] Douglas E.A., Reza S., Sanchez C., Koleske D., Allerman A., Klein B., Armstrong A.M., Kaplar R.J., Baca A.G. (2017). Ohmic contacts to Al-rich AlGaN heterostructures. Phys. Status Solidi (A).

[29] Wang L., Kim D.H., Adesida I. (2009). Direct contact mechanism of Ohmic metallization to AlGaN/GaN heterostructures via Ohmic area recess etching. Appl. Phys. Lett..

[30] Lu H., Hou B., Yang L., Song F., Zhang M., Wu M., Ma X., Hao Y. (2022). Low-resistance Ta/Al/Ni/Au ohmic contact and formation mechanism on AlN/GaN HEMT. IEEE Trans. Electron Devices.

[31] Yang L., Lu H., Niu X., Zhang M., Shi C., Deng L., Hou B., Mi M., Wu M., Cheng K. (2022). Investigation of contact mechanism and gate electrostatic control in multi-channel AlGaN/GaN high electron mobility transistors with deep recessed ohmic contact. J. Appl. Phys..

[32] Guo H., Shao P., Zeng C., Bai H., Wang R., Pan D., Chen P., Chen D., Lu H., Zhang R. (2022). Improved LPCVD-SiNx/AlGaN/GaN MIS-HEMTs by using in-situ MOCVD-SiNx as an interface sacrificial layer. Appl. Surf. Sci..

[33] Liu X., Wang X., Zhang Y., Wei K., Zheng Y., Kang X., Jiang H., Li J., Wang W., Wu X. (2018). Insight into the near-conduction band states at the crystallized interface between GaN and SiN x grown by low-pressure chemical vapor deposition. ACS Appl. Mater. Interfaces.

[34] Lu H., Ma X., Hou B., Yang L., Zhang M., Wu M., Si Z., Zhang X., Niu X., Hao Y. (2021). Improved RF power performance of AlGaN/GaN HEMT using by Ti/Au/Al/Ni/Au shallow trench etching ohmic contact. IEEE Trans. Electron Devices.

[35] Jimenez J.L., Chowdhury U. X-Band GaN FET reliability. Proceedings of the 2008 IEEE International Reliability Physics Symposium, 2008.

[36] Chen D.Y., Persson A.R., Wen K.H., Sommer D., Grünenpütt J., Blanck H., Thorsell M., Kordina O., Darakchieva V., Rorsman N. (2022). Impact of in situ NH3 pre-treatment of LPCVD SiN passivation on GaN HEMT performance. Semicond. Sci. Technol..

[37] Huang T., Bergsten J., Thorsell M., Rorsman N. (2018). Small-and large-signal analyses of different low-pressure-chemical-vapor-deposition SiN x passivations for microwave GaN HEMTs. IEEE Trans. Electron Devices.

[38] Jing G., Wang X., Huang S., Jiang Q., Deng K., Wang Y., Li Y., Fan J., Wei K., Liu X. (2022). Mechanism of Linearity Improvement in GaN HEMTs by Low Pressure Chemical Vapor Deposition-SiN x Passivation. IEEE Trans. Electron Devices.

